# Nordic hip fracture registries: comparisons toward a minimal common and standardized Nordic Hip Fracture Dataset (Nordic-HFD)

**DOI:** 10.2340/17453674.2026.46720

**Published:** 2026-09-03

**Authors:** Christian R SCHMIDT, Henrik PALM, Bjarke VIBERG, Margareta HEDSTRÖM, Olof WOLF, Cecilia ROGMARK, Jan-Erik GJERTSEN, Eva DYBVIK, Alma B PEDERSEN

**Affiliations:** 1 Department of Clinical Epidemiology, Aarhus University Hospital and Aarhus University, Aarhus, Denmark; 2 Department of Orthopaedic Surgery, Copenhagen University Hospital, Bispebjerg, Copenhagen, Denmark; 3 Department of Orthopaedic Surgery and Traumatology, Odense University Hospital, Odense, Denmark; 4 Department of Clinical Science, Intervention and Technology (CLINTEC), Karolinska Institutet, Sweden and The Swedish National Registry for hip fractures, Trauma and Reparative Medicine Theme (TRM), Karolinska University Hospital, Stockholm, Sweden; 5 Department of Surgical Sciences, Orthopaedics, Uppsala University, Uppsala, Sweden; 6 Department of Orthopaedics, Lund University, Skåne University Hospital, Malmö, Sweden; 7 Norwegian Hip Fracture Register, Department of Orthopaedic Surgery, Haukeland University Hospital, Bergen, Norway; 8 Department of Clinical Medicine, University of Bergen, Bergen, Norway

## Abstract

**Background and purpose:**

National hip fracture registries in Denmark, Norway, and Sweden collect extensive pre- and post-fracture data. Inspired by the Global Fragility Fracture Network (Global FFN) minimum common dataset, which is not specifically tailored to Nordic healthcare systems, we aimed to evaluate the feasibility of developing a standardized Nordic Hip Fracture Dataset (Nordic-HFD).

**Methods:**

We compared 4 Nordic hip fracture registries: the Danish Multidisciplinary Hip Fracture Registry, the Norwegian Hip Fracture Register, the Swedish Fracture Register, and the Swedish Hip Fracture Register (RIKSHÖFT). Variables available in at least 3 registries, including those obtainable through linkage with national health registries, were assessed for definitions and grouped into demographic, surgical, postoperative, complications, and PROM domains.

**Results:**

Across registries, 38 variables were identified. The number of variables available in all 4 registries was 11, while 31 were available in at least 3 registries. After quality review, 30 variables were ultimately considered suitable for inclusion in the proposed Nordic-HFD.

Demographic variables such as age, sex, fracture type, pathological fracture, laterality, ASA score, surgical variables such as surgical method, date of surgery, time to surgery, and mortality were directly comparable. Variables on mobility, cognitive impairment, complications, and PROMs may require further harmonization.

**Conclusion:**

Establishing a Nordic-HFD is feasible using existing national registries’ infrastructures. The remaining challenge is harmonizing variable definitions to specific research questions and future validation studies. A Nordic-HFD would enhance robust cross-country benchmarking and support international initiatives such as the Global FFN.

Hip fractures remain a major global healthcare challenge, associated with high morbidity, mortality, and economic burden [1]. To enhance the quality of patient care, treatment, and outcomes of patients with a hip fracture, national registries have been established during the past decades. However, these registries vary considerably in organization, methodology, and the type of data collected, including outcomes [2]. To overcome this, an international attempt has been made to create a global Minimum Common Dataset (MCD). In 2022, the Global Fragility Fracture Network (Global FFN) proposed a revised MCD [3]. However, the Global FFN MCD is not designed specifically according to the Nordic healthcare systems and their existing national hip fracture registries, which are established mainly by orthopedic surgeons.

The Nordic countries of Denmark, Norway, and Sweden have similar healthcare systems and a long tradition of hip fracture registries, with hip fracture incidences exceeding most other countries [4]. Although each country comprises large cohorts, individual national registries may lack statistical power to analyze some subgroups of fracture population [5]. With a combined total population of over 22,000,000 individuals and relatively uniform cohorts and healthcare systems, Nordic collaboration could be an option to strengthen the statistical power of epidemiological analyses. Nordic arthroplasty registries have cooperated within the Nordic Arthroplasty Register Association (NARA) since 2007, establishing a set of common variables, linking data from all national arthroplasty registries into 1 common dataset, and contributing with a number of pieces of quality and research evidence [6-8]. In light of the successful cooperation within NARA, it would be a natural development to establish a similar collaboration between the Nordic hip fracture registries.

We therefore aim to compare the Danish, Norwegian, and Swedish hip fracture registries and explore the feasibility of developing a standardized Nordic Hip Fracture Dataset (Nordic-HFD), with the ultimate goal of improving quality of treatment and safety of hip fracture patients.

## Methods

### Study design

This study is descriptive using prospectively collected data from 4 established hip fracture registries in Denmark (n = 1), Norway (n = 1), and Sweden (n = 2). The study was conducted according to the STROBE and RECORD guidelines.

### Study setting

Denmark, Norway, and Sweden share a publicly funded, tax-based healthcare model that provides universal access to primary and secondary care, at no or very low cost [9]. These systems are characterized by comprehensive national coverage, standardized care pathways, and a substantial amount of data being collected in a centralized infrastructure. Despite some administrative differences, the overall structure ensures that nearly all citizens receive acute medical care within a unified public system, enabling consistent and population-wide data collection across both primary and secondary care institutions [9,10]. Another defining feature of the Nordic healthcare model is unique personal identification numbers (PINs), given to all residents at birth, that enable individual-level and long-term follow-up of patients passing through different institutions. Further, PINs allow linkage across virtually all national health and administrative registries, such as patient registries, prescription databases, death registries, birth registries, and disease-specific quality registries, despite sometimes being time consuming because of legislation and application processes [9-11].

### Data sources

#### Hip fracture registries

The study comprises the Danish Multidisciplinary Hip Fracture Registry (DMHFR), the Norwegian Hip Fracture Registry (NHFR), the Swedish Fracture Registry (SFR), and the Swedish Hip Fracture Register (RIKSHÖFT) (Table 1)

**Table 1:** Key characteristics and numbers regarding the respective hip fracture registries

Factor	DMHFR ^a^	NHFR ^a^	SFR ^a^	RIKSHÖFT ^b^
Country	Denmark	Norway	Sweden	Sweden
Year established	2003	2005	2011 **^c^**	1988
Primary hip fractures registered	> 128,000	> 163,000	> 155,000	> 350,000
National hospital coverage	All	All	All	38 of 52 **^d^**
Patient completeness, primary surgery	~100%	Osteosynthesis 82%, HA 91%, THA 88%	~85%	> 80% **^e^**
Inclusion criteria (study population)				
Age group	> 65 years	All ages	All ages	> 15 years
Treatment for hip fracture	Surgical	Surgical	Surgical and non-surgical	Surgical and non-surgical
Data collection	Clinical registry (web-based)	Clinical registry (web-based)	Clinical registry (web-based)	Clinical registry (web-based)
Linkage possibility **^f^**	Yes	Yes	Yes	Yes

DMHFR = Danish Multidisciplinary Hip Fracture Registry, NHFR = Norwegian Hip Fracture Register, SFR = Swedish Fracture Registry, HA = hemiarthroplasty, THA = total hip arthroplasty.

a Table data from respected registry year reports and online statistics, from 2024 and 2025.

b RIKSHÖFT has been paused since summer 2025. Table data from prior years.

c Hip fracture data from 2012.

d In 2023.

e From 2008–2020.

f Linkage possibility to all administrative registries (to obtain data on hospitalizations, comorbidities, other surgeries, prescriptions, laboratory and microbiology tests, mortality, cause of death, socioeconomic status, social services, etc.) and clinical quality databases (to obtain data on other fractures, surgical procedures, etc.) using unique civil registration number and after ethical approval.

The DMHFR was established in 2003, as part of the umbrella organization Danish Clinical Registries, and consists of a multidisciplinary steering committee [12]. Reporting to DMHFR has been mandatory by law for all Danish hospitals since 2006, resulting in high completeness within the inclusion criteria, though exact completeness is unknown [13]. As of 2025, DMHFR has data on more than 128,000 hip fractures [12]. Healthcare professionals involved in the treatment of patients with hip fractures report prospectively to DMHFR during hospitalization. Data is collected on all patients above 65 years of age who have been surgically treated for hip fracture [14].

The NHFR was established in 2005 and has a completeness of 82% for osteosyntheses, 91% for hemiarthroplasties, and 88% for total hip arthroplasties. As of 2025, the NHFR has data on more than 163,000 primary hip fractures [15]. All Norwegian hospitals managing hip fractures report to the registry, and data collection is eligible for all surgically treated hip fracture patients, regardless of age.

The SFR was established in 2011 with tibia and humerus fracture registration. Since April 2012, patients of any age, with any type of fracture, surgically or non-surgically treated, have been eligible for inclusion. It has gradually progressed in size over the last decade, and as of 2021 all orthopedic and trauma departments in Sweden have reported to this database. The completeness of registrations in 2024 of femur fractures was 85%. As of 2025, the accumulated number of registered fractures reached more than 1 million, with more than 155,000 hip fractures being included [16-18].

The RIKSHÖFT, the original Swedish national registry of hip fractures, was established in 1988 as the world’s first national database on patients with hip fractures. Data has been collected on all patients above 15 years of age, surgically or non-surgically treated for hip fracture. RIKSHÖFT has been paused since the summer of 2025; however, all previously collected data, on more than 350,000 hip fractures, is available [19]. Completeness of primary hip fractures compared with the Swedish national patient register was 81% in the period 2008–2017 and as of 2023 hospital coverage was 38 of 52 hospitals managing hip fractures [20,21].

#### Other data sources

All 4 registries, to varying degrees, rely on data from other national sources. For example, data from the Norwegian Arthroplasty Registry (on total hip arthroplasty) and Swedish Arthroplasty Registry (on hemi- and total hip arthroplasty) are copied/co-processed into NHFR and SFR, respectively. In Denmark, all data on hip fractures is for quality and research purposes linked to the Danish National Patient Registry, the Danish National Prescription Registry, and other data sources.

### Statistics

We systematically mapped all variables available in the 4 hip fracture registries. We further assessed whether missing variables in the hip fracture registries were easily obtainable from other national registries, to sum up overall availability for each variable. The variables, which in some registries were only obtainable from alternative national databases, we considered “fulfilled data points” and are described as being an available variable in our cross-registry comparison. For each variable, we determined the timeframe of data collection, definition, categorization, and comparability.

When aiming to identify potential common variables for a future Nordic-HFD, we prioritized those available in at least 3 registries. Variables with lower availability were excluded due to insufficient cross-country comparability (Figure).

**Figure:**
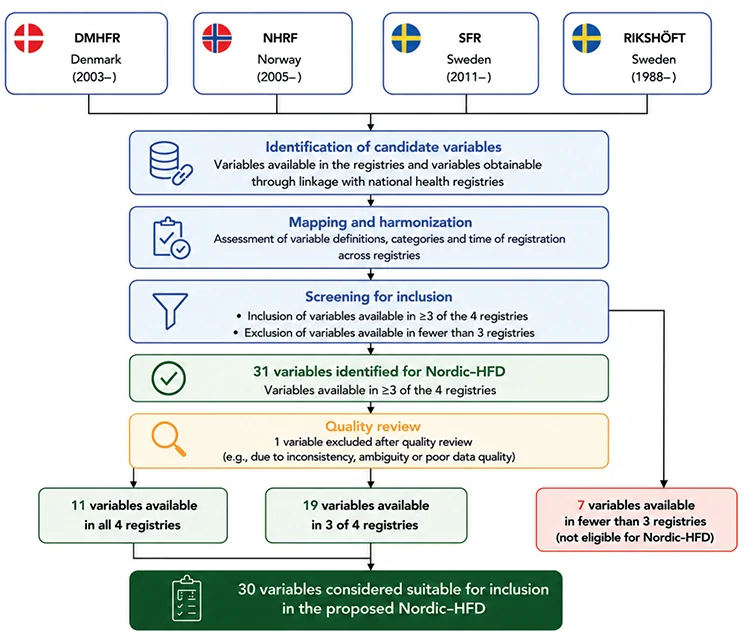
Study flow diagram: from registry identification to Nordic-HFD candidate variables.

### Ethics, data-sharing, use of AI, funding, and disclosures

The study was reported to the Danish Data Protection Agency through registration at Aarhus University (record number AU-2016-051-000001, number 880). Due to Nordic legislation, no further data sharing is possible. AI-based tools were used solely to assist with grammatical and punctuation improvements, as well as figure modification. Authors received no external funding for this study. JEG has received speaker fees from Smith & Nephew, Heraeus Medical, and Ortomedic (Norwegian manufacturer for DePuy Synthes). BV has received a research grant from Swemac. MH has received lecture fees from DePuy Synthes and Johnson & Johnson for lectures and a total knee arthroplasty course in 2024 and 2025. OW has received lecture fees from LINK Sweden, Smith & Nephew, DePuy Synthes, Swemac, participation fees for DSMC for Fruiti and Lit trial—2023, DSMC Initiate 2024—and has a leadership role as Director of the Swedish Fracture Register, and Chair of the Swedish Orthopaedic Trauma Society. CR has received lecture fees from Swemac and LINK, and has a leadership role in Acta Orthopaedica as co-editor. CRS, HP, ABP, and ED have no disclosures. Complete disclosure of interest forms according to ICMJE are available on the article page, doi: 10.2340/17453674.2026.46720

## Results

### Variable availability

38 candidate variables were identified across the 4 registries. These variables were grouped into “Demographics,” “Operative,” “Postoperative,” “Complication-related,” and “PROM” domains (Table 2). The candidate variables were then evaluated according to their availability (Table 3).

**Table 2:** Year of beginning of registration for each candidate variable, sorted by category, availability of variables and selected potential Nordic-HFD variables

Factor	DMHFR	NHFR	SFR ^a^	RIKS-HÖFT ^b^	Availability	Potential Nordic-HFD variables
Demographics						
Age	2006	2005	2011	1988	4	Yes
Sex	2006	2005	2011	1988	4	Yes
Fracture diagnosis	2006	2005	2011	1988	4	Yes
Pathological fracture	2006	2005	2015	1998	4	Yes
Fracture displacement	2006	2005	2011	1988	4	Yes
Laterality	2010	2005	2011	1988	4	Yes
ASA score	2006	2005	2011	1988	4	Yes
Pre-fracture						
cognitive impairment	2022	2005	–	2006	3	Yes
cohabitation	2006	2023	–	1988	3	Yes
anticoagulants	2006	2011	–	2014 **^c^**	3	Yes
mobility	2006	2023	–	1988	3	Yes
CCI	2006	2005	–	–	2	
Body mass index	2006	–	–	2013	2	
Surgery						
Primary surgical method	2006	2005	2011	1988	4	Yes
Date of primary surgery	2006	2005	2015 **^d^**	1998 **^e^**	4	Yes
Time to surgery	2006	2005	2015	1998	4	Yes
Mortality	2006	2005	2011	1988	4	Yes
Reoperation	2006	2005	2011	–	3	Yes
Date of reoperation	2006	2005	2011	–	3	Yes
Cause of reoperation	2010	2005	2011	–	3	Yes
Non-surgical treatment	2006	–	2011	1988	3	Yes
Duration of surgery	2006	2005	–	1998	3	Yes
Preoperative optimization	2015	–	–	–	1	
Post surgery						
Discharge destination	2010	2023	–	2010	3	Yes
Early mobilization	2010	2023	–	Unknown	3	Yes
Osteoporosis prevention	2006	2023	–	2010 f	3	Yes
(Mal)Nutrition screening	2006	2023	–	–	2	
Fall prevention	2010	2023	–	–	2	
Mobility at discharge	2006	–	–	–	1	
Complications						
Pressure ulcer/decubitus	2006	2023	–	1999	3	Yes
Urinary tract infection	2006	2023	–	1999	3	Yes
Pneumonia	2006	2023	–	1999	3	Yes
Acute kidney injury	2006	2023	–	1999	3	Yes
Thrombosis	2006	2023	–	1999	3	Yes
Cardiovascular event	2006	2023	–	1999	3	Yes
Delirium	2022	2023	–	1999	3	Yes
PROM						
At baseline	–	2005	2011	–	2	
Follow-up **^g^**	–	2005	2011	2010 **^f^**	3	

ASA = American Society of Anesthesiologists; CCI = Charlson Comorbidity Index; DMHFR = Danish Multidisciplinary Hip Fracture Registry; NHFR = Norwegian Hip Fracture Register; SFR = Swedish Fracture Registry.

“Unknown”: year of variable application not fully known/clearly defined.

a Hip fractures from 2012.

b RIKSHÖFT has been paused since summer 2025. Table data from prior years.

c Yes/no from 2004. Type from 2014.

d Date from 2012. Time from 2015.

e Date from 1988. Time from 1998.

f Incomplete registration.

g Excluded as potential Nordic-HFD variable after quality review.

**Table 3:** Availability of candidate variables across registries. Values are count

Domain	Total no of candidate variables	Number of variables available in				Suitable for inclusion in Nordic-HFD (≥ 3 registries)
		all 4 registries	3 of 4 registries	2 of 4 registries	1 of 4 registries	
Demographics	13	7	4	2	0	11
Surgery	10	4	5	0	1	9
Post-surgery	6	0	3	2	1	3
Complications	7	0	7	0	0	7
PROM	2	0	(1) **^a^**	1	0	0
Total	38	11	19	5	2	30 **^b^**

a The 1 patient-reported outcome (PROM) variable identified as suitable (available in ≥ 3 registries) was excluded after quality review due to insufficient data completeness and/or non-comparable definitions across registries.

b Final number of variables for inclusion in Nordic-HFD after quality review: 30 (of 38), see Table 2.

11 variables were available in all 4 registries, including “age,” “sex,” “fracture diagnosis,” “pathological fracture,” “fracture displacement,” “laterality,” “American Society of Anesthesiologists score,” “primary surgical method,” “date of surgery,” “time to surgery,” and “mortality” (Table S1, see Supplementary data).

An additional 20 variables were available in 3 out of 4 registries, including “pre-fracture cognitive impairment,” “pre-fracture cohabitation,” “pre-fracture anticoagulants,” “pre-fracture mobility,” “reoperation,” “date of reoperation,” “cause of reoperation,” “non-surgical treatment,” “duration of surgery,” “discharge destination,” “early mobilization,” “osteoporosis prevention,” “pressure ulcer/decubitus,” “urinary tract infection,” “pneumonia,” “acute kidney injury,” “thrombosis,” “cardiovascular event,” “delirium,” and “PROM” (Table S2, see Supplementary data).

From the 38 candidate variables, 30 variables were deemed suitable for inclusion in a Nordic-HFD (Table 3).

## Discussion

We aimed to evaluate the feasibility of developing a standardized Nordic-HFD. We found that the Danish, Norwegian, and Swedish hip fracture registries already share a substantial common data structure, supporting both the feasibility and the clinical relevance of developing a unified Nordic-HFD. Across the 4 registries, 30 variables were found suitable for inclusion. Of these, 11 variables were consistently available in all registries, encompassing core demographic data, fracture characteristics, key surgical data, and mortality outcome.

### Comparability of variables in Nordic-HFD

Overall, the variables included in a Nordic-HFD have good potential for comparison. Core variables such as age, sex, laterality, and dates of surgery and death are simple, consistently collected, and uniformly defined across all registries. Other variables can be compared through reclassification into broader, simplified, and harmonized categories. For example, “fracture diagnosis” is coded using AO/OTA classification or Garden subclasses in some registries and ICD-based categories in others. However, all registries ultimately distinguish 3 major anatomical groups: femoral neck, trochanteric, and subtrochanteric fractures. Collapsing registry-specific classifications into these shared groups enables valid cross-country comparison, although some granularity such as fracture displacement or Garden staging is necessarily lost. Likewise, “early mobilization” can be aligned by simplifying definitions such as “mobilization within the 1st or 2nd postoperative day,” recognizing that exact time from surgery to mobilization may be lost.

Some variables show varying degrees of structural differences across registries, which limits the possibility of straightforward reclassification. A clear example is “pre-fracture mobility.” The DMHFR employs the “Cumulated Ambulation Score,” which integrates assessments of getting in and out of bed, sitting-to-standing-to-sitting from a chair, and walking. In contrast, the NHFR and RIKSHÖFT record only walking ability and use of walking aids, each with its own categorical groupings. Valid cross-registry comparison would thus require substantial harmonization. Another example is “acute kidney injury.” The NHFR defines it solely through increases in serum creatinine, RIKSHÖFT through elevations in either creatinine or urea, and DMHFR technically through any laboratory data threshold. As a result, cross-registry comparisons would likely be misleading unless definitions were standardized or supported by scientific studies showing high compatibility of specific laboratory results. Regarding “pre-fracture cohabitation” and “discharge destination,” creating common datapoints such as “home” or “institution” would provide simplified but clinically meaningful distinctions.

### Coverage, completeness, and validity of Nordic-HFD

Although national coverage in the Nordic hip fracture registries generally is high, completeness may vary across variables, time periods, and individual registries. Differences in start dates for variable implementation may lead to inequalities in follow-up periods, duration, and smaller study populations for selected measures. These inconsistencies can limit longitudinal comparisons and reduce statistical power, particularly for variables introduced in later periods. Furthermore, inclusion of variables obtained through linkage with external national registries relies on continuous and consistent data access, which may be influenced by administrative and legislative factors.

Except for coverage and completeness of registration, the validity of registered variables is crucial for research quality and interpretation. Several validation studies support the robustness of the Nordic registries. In the DMHFR, the positive predictive value of hip fracture diagnoses and procedure codes exceeds 90% when compared with gold-standard medical records [22]. Validated algorithms for identifying reoperations have also been established in Denmark [23]. The SFR has showed high agreement between registry-recorded AO/OTA classification and experts’ adjudication [24]. The RIKSHÖFT has reported 93% agreement in hip fracture classifications when compared with the national patient registry [20]. The accuracy of cognitive function assessment has been evaluated in the NHFR. The assessment of cognitive impairment by orthopedic surgeons had a positive predictive value of 78% and a negative predictive value of 84% when compared with quality databases from 2 hospitals [25]. In another study, the ability of orthopedic surgeons to detect pathological fractures was examined; among 1,484 suspected cases, 485 were not pathological fractures [26].

An additional challenge for a future Nordic-HFD is the temporary suspension of the RIKSHÖFT as of summer 2025, with no expected timeline for reopening. For variables currently available in 3 of the 4 registries—where the RIKSHÖFT constitutes one of the contributors—revised harmonization strategies may be required. Possible options include removal of these variables from the Nordic‑HFD, adoption of variables by another national registry, or acceptance of variables present in only 2 of 3 active registries. Nevertheless, the RIKSHÖFT data remains fully available up to and including 2024, ensuring early Nordic‑HFD integration.

Together, these findings indicate that the Nordic hip fracture registries provide high quality data suitable for research and benchmarking, while also highlighting the importance of ongoing efforts to ensure systematic validation of data, consistency in variable completeness, and follow up.

### Interpretation

The proposed Nordic-HFD aligns closely with the recently updated Global FFN MCD, which emphasizes standardized variables to support international benchmarking [27]. These core variables, including demographics, fracture classification, surgical details, mobility status, and key outcomes, are already well represented across the Nordic hip fracture registries. In several respects, the Nordic-HFD would exceed Global FFN MCD with a broader range of core and outcome variables. Our findings also mirror challenges described by Global FFN. International comparability depends on simplified and standardized variable definition to minimize misclassification and enhance reproducibility. The same principle applies for a future Nordic-HFD. Despite substantial similarities between the registries, variation remains in the definitions and granularity of several variables. Effective cross‑country comparison will therefore require adoption of harmonized, simplified definitions. Hopefully, introduction of a Nordic-HFD can help facilitate this, with a recent study showing 17 hip fracture registries in 20 countries improving compatibility after introduction of the Global FFN MCD in 2022 [28].

Given the shared characteristics of Nordic healthcare systems and their hip fracture populations, a Nordic-HFD built upon existing registry infrastructure and broadly aligned with the Global FFN MCD holds great potential for advancing patient care [29]. While the Global FFN MCD provides the minimum requirements for international comparability, the Nordic registries contain several additional variables that may help identify and address important knowledge gaps in fracture care. In this context, both the Global FFN MCD and the Nordic HFD may serve as practical models for implementation in countries without government-driven or tax-funded registry systems, where establishment of hip fracture registries initially may rely on voluntary participation coordinated by national orthopedic, geriatric, or multidisciplinary fracture societies.

### Conclusion

Our study shows that developing a Nordic-HFD is feasible with existing registry infrastructures. Following this comprehensive mapping of current registries, the next step is to agree on simplified, standardized variable groupings to support a shared dataset and robust cross country comparison in line with specific research questions and future validation studies. A unified Nordic-HFD would strengthen evidence-based research and enhance quality improvement efforts in hip-fracture care across the Nordic countries and globally [30].

### Supplementary data

Supplementary Tables S1–S2 are available as supplementary data on the article page, doi: 10.2340/17453674.2026.46720

All authors contributed to the study design, interpretation of the results, and edited the manuscript. CRS wrote the first draft of the manuscript in collaboration with ABP. All authors had final responsibility for the decision to submit for publication.

## Supplementary Material



## Notes

Handling co-editors: Keijo Mäkelä and Robin Christensen

*Acta* thanks Enrico De Visser and Martyn Parker for help with peer review of this manuscript.
