# Supplementary material for: Nordic hip fracture registries: comparisons toward a minimal common and standardized Nordic Hip Fracture Dataset (Nordic-HFD)
Source: Acta Orthop. 2026 Sep 3;97:595–601. doi: 10.2340/17453674.2026.46720 (PMC13543152; doi:10.2340/17453674.2026.46720)
Supplement: Supplementary file 1 [file ActaO-97-46720-s1.pdf]

Supplementary material: Nordic hip fracture registries - comparisons toward a minimal common and standardized Nordic Hip Fracture Dataset (Nordic-HFD)

Supplement Table S1. Variables that are available in all 4 registries, with underlying variable definitions and data points.

	DMHFR	NHFR	SFR	RIKSHÖFT
**Baseline**				
**Age at fracture**	Years	Years	Years	Years
**Sex**	Male/Female	Male/Female	Male/Female	Male/Female
**Fracture diagnosis**	ICD-10 codes:<br>* S72.0: (femoral neck).<br>* S72.1: pertrochanteric.<br>* S72.2: subtrochanteric.	Based on Garden and AO/OTA classification:<br>* non-displaced femoral neck (Garden I–II).<br>* displaced femoral neck (Garden III–IV),<br>* Baso cervical.<br>* 2-fragment pertrochanteric (AO/OTA A1).<br>* multi-fragment pertrochanteric (AO/OTA A2).<br>* intertrochanteric (AO/OTA A3).<br>* subtrochanteric fracture.	ICD-10 codes:<br>* S72.00: closed femoral neck.<br>* S72.01: open femoral neck.<br>* S72.10: closed pertrochanteric.<br>* S72.11: open pertrochanteric.<br>* S72.20: closed subtrochanteric.<br>* S72.21: open subtrochanteric.<br>Additionally classified according to AO/OTA (A1–3, B1–3).<br><br>Periprosthetic fractures according to UCS-systems	* non-displaced femoral neck (Garden I–II).<br>* displaced femoral neck (Garden III–IV).<br>* basocervical.<br>* 2-fragment trochanteric.<br>* multi-fragment trochanteric.<br>* subtrochanteric.
**Pathological fracture**	ICD-10 code:<br>* M84.4 pathological fracture.	* non-pathological fracture.<br>* pathological fracture.<br>* uncertain.	ICD-10 code:<br>* M84.4 pathological fracture.	* non-pathological fracture.<br>* metastatic fracture.<br>* benign bone tumor.<br>* primary malign bone tumor.<br>* Paget.<br>* other.
**Fracture displacement**	Supplementary diagnostic codes added to fracture codes S72.0–S72.2:<br>* ZDS01: displaced fracture.<br>* ZDS02: non-displaced fracture.<br>* ZDS03: unknown displacement.	Femoral neck fractures classified as undisplaced (Garden 1+2) or displaced (Garden 3-4).<br>From 2021:<br>Posterior tilt (< or > 20 degrees) are registered for Garden 1+2 fractures.	Classified according to AO/OTA (A1–3, B1–3).	Additional question, marked “yes” for either non-dislocated or dislocated.
**Laterality**	* right side.<br>* left side.	* right side.<br>* left side.	* right side.<br>* left side.	* right side.<br>* left side.

	* missing information.			
**American Society of Anesthesiologists score**	Available from Danish Anesthesiologic Database	Available from fracture register	Available from Swedish Perioperative Register	Available from fracture register
**Surgery**				
**Primary surgical method**	Procedure codes:<br>* KNFB0–99: primary insertion of hip arthroplasty.<br>* KNFJ4–9: fracture treatment of femur (osteosynthesis).<br>* KNFG0–99: resection arthroplasty of the hip joint.<br>* KNFK19: resection or excision of bone in femur.<br>These codes are grouped into main categories (e.g. osteosynthesis, hemiarthroplasty, THA).	* 2 screws or pins.<br>* 3 screws or pins,<br>* bipolar hemiarthroplasty.<br>* unipolar hemiarthroplasty.<br>* sliding hip screw and plate.<br>* sliding hip screw with trochanteric support plate.<br>* short intramedullary nail without distal locking.<br>* short intramedullary nail with distal locking.<br>* long intramedullary nail without distal locking.<br>* long intramedullary nail with distal locking.<br>* other procedure.<br>* total hip arthroplasty.	Procedure codes:<br>* NFB09–99: primary insertion of hip arthroplasty.<br>* NFJ09–99: fracture treatment of femur.<br>* NFG09–99: resection arthroplasty in the hip joint.<br>* NFK19: resection or excision of femoral bone.<br>* TNF39.0: Non-operative treatment.<br>* TNX40: Traction without anesthesia.	* one screw, pin or nail.<br>* 2 screws, pins or nails.<br>* 3 screws, pins or nails.<br>* screw, pin or nail with side-plate.<br>* intramedullary nail.<br>* hemiarthroplasty (half/bipolar).<br>* total hip arthroplasty.<br>* conservative (non-surgical) treatment.<br>* other treatment.
**Date of primary surgery**	* Date and time	* Date and time	* Date and time	* Date and time
**Time to surgery**	Calculated from exact date and time of hospital admission and surgery, available from the National Patient Registry.	Counted from time of fracture as following.<br>* 0–6 hours.<br>* >6–12 hours.<br>* >12–24 hours.<br>* >24–48 hours.<br>* > 48 hours.<br>Since 2023 also time from admission available	Available as continuous variable (hours & minutes) from time of X-ray to start of surgical procedure. Reported as proportions of hip fracture patients ≥20 years operated:<br>* within 24 hours of X-ray diagnosis.<br>* within 36 hours of X-ray diagnosis.	Recorded both as exact time from admission to surgery and as 2 categories:<br>* operation within 24 hours.<br>* operation within 36 hours.
**Mortality**	Date of death at any time after surgery obtained from national registries; used to calculate postoperative mortality.	Date of death at any time after surgery obtained from national population register; used to calculate postoperative mortality.	Date of death at any time after fracture obtained from national registries; used to calculate post fracture mortality.	Registered as mortality after 4 month and 12 months. Post operative

DMHFR = Danish Multidisciplinary Hip Fracture Registry, NHFR = Norwegian Hip Fracture Register, SFR = Swedish Fracture Registry.

Supplement Table S2: Variables that are available in 3 out of the 4 registries, with underlying variable definitions and data points.

	DMHFR	NHFR	SFR	RIKSHÖFT
**Baseline**				
**Pre-fracture cognitive impairment**	Derived from diagnostic codes and a cognitive assessment code (ZZ5061). Results coded as:<br>* ZRPB00: cognitive impairment.<br>* ZRPB01: not cognitively impaired.<br>* ZRPB02: unknown cognitive status.	* Yes.<br>* No.<br>* Uncertain.	-	* No cognitive impairment.<br>* Suspected dementia/confusion.<br>* Diagnosed with dementia.
**Pre-fracture cohabitation**	Derived from diagnostic code ZZ8050 with additional codes:<br>* ZRSB01: living in own home.<br>* ZRSB01A: living in own home affiliated to an institution.<br>* ZRSB02: homeless.<br>* ZRSB04: living in residential care facility/nursing home.<br>* ZRSB09: no information available.	* living in own home.<br>* living in institution.<br>* from acute care (other hospital ward).<br>* staying at rehabilitation facility.<br>* other.	-	* living in own home.<br>* living in residential care facility.<br>* living in own home at a residential care facility.<br>* staying at rehabilitation facility.<br>* from acute department.<br>* other.
**Pre-fracture mobility**	Cumulated Ambulation Score (CAS).<br>Score 1-6 and unknown.<br>Include assessment of: Getting in and out of bed, sit-to-stand-to-sit from a chair, indoor walking.<br>Each activity is assessed on scale from 0-2 (0 = Not able, 1 = Able to with assistance, 2 = Able to safely, without assistance).	Scale 1-6:<br>* Freely mobile without aids.<br>* Mobile outdoor with one aid.<br>* Mobile outdoor with 2 aids or frame.<br>* Some indoor mobility, but never goes outdoor without help.<br>* No functional mobility.<br>* Unknown.	-	2 separate 1–5 scales.<br><br>Walking:<br>* Walked outdoors alone.<br>* Walked outdoors only with company.<br>* Walked indoors alone but not outdoors.<br>* Walked outdoors only with company.<br>* Unable to walk.<br><br>Mobility aid:<br>* No walking aids.<br>* One cane.<br>* 2 canes.<br>* Rollator walker.

				* Wheelchair or never left bed.
**Pre-fracture anti-coagulants**	Available from the National Prescription Registry.	3 main outcomes<br>* Yes<br>* No.<br>* Unknown.<br>Medication type also recorded.	-	Recorded as yes/no to use of anticoagulant medication. Type since 2015
**Surgery**				
**Reoperation**	From procedure codes: KNFU0-99 combined with<br>* NFB0-99: Primary prosthetic replacement of hip joint.<br>* NFC0-99: Secondary prosthetic replacement of hip joint.<br>* NFW00-69: Reoperation on hip or thigh.<br>* NFG09: excision arthroplasty of hip joint<br>* NFU0-99: Removal of implants and external fixation devices from hip and femur.<br>* NFS0-99: Operation for infection of tendon, joints and bone of hip and thigh.	* Removal of implant.<br>* Girdlestone.<br>* Bipolar hemiarthroplasty.<br>* Unipolar hemiarthroplasty.<br>* Infection debridement.<br>* Closed reduction of dislocated. arthroplasty.<br>* Open reduction of dislocated. arthroplasty.<br>* Exchange caput / bipolar head.<br>* Other.<br>* THA information available from NAR	Registered but completeness unknown. Information exchanged with Swedish Arthroplasty Register on arthroplasty procedures on fracture patients, and fractures on arthroplasty patients	Registered but completeness unknown. Not validated against the National Patient Register
**Date of reoperation**	* Date and time	* Date and time	* Date and time	-
**Cause of reoperation**	Available from the National Patient Registry using diagnostic codes, indicating causes such as:<br>infection, dislocation, pseudoarthrosis, mechanical arthroplasty complication, bleeding, wound rupture, new fracture etc.	* osteosynthesis failure,<br>* non-union.<br>* avascular necrosis of the femoral head.<br>* local pain due to prominent osteosynthesis material.<br>* malunion.<br>* prolonged wound drainage.<br>* superficial wound infection.<br>* deep wound infection.<br>* hematoma,<br>* dislocation of hemiarthroplasty.<br>* Cutout of osteosynthesis material.	Registered with detailed description (patient symptoms, infection, hardware failure, nonunion etc)	-

		* peri-implant fracture.<br>* loosening of hemi-arthroplasty.<br>* other.<br>* THA information available from NAR		
**Non-surgical treatment**	Available from the National Patient Registry	-	Available from fracture registry	Available from fracture registry
**Duration of surgery**	Exact time available	Exact time available		Exact time available
**Post-surgery**				
**Discharge destination**	Derived from code ZZ8050 with discharge destination codes:<br>* ZRSB01: own home.<br>* ZRSB01A: own home affiliated to an institution.<br>* ZRSB02: homeless.<br>* ZRSB04: residential care facility/nursing home.<br>* ZRSB09: no information.<br>These are often grouped as home, institution, rehabilitation, other.	* Own home.<br>* Institution.<br>* Other hospital or department.<br>* Rehabilitation facility.<br>* Other.	-	At 4 month:<br>* own home.<br>* residential care facility.<br>* own home at residential care facility.<br>* rehabilitation facility.<br>* acute/emergency department.<br>* other.<br>* death.
**Early mobilization**	Exact date and time of first postoperative mobilization are registered, allowing calculation of time from surgery to first mobilization.	* not mobilized on postoperative day 1.<br>* mobilized on postoperative day 1.<br>* unknown	-	Exact number of days from surgery to first mobilization is recorded.
**Osteoporosis prevention**	Available from fracture registry and the National Prescription Registry	Information on type of osteoporosis prevention.<br>For each type of medication:<br>* not given.<br>* Initiated.<br>* continued.<br>* unknown	-	Information on type of osteoporosis prevention.<br>For each type of medication, information available on start and duration of treatment.
**Complications**				
**Pressure ulcer/decubitus**	Available through ICD-10 code from the National Patient Registry:<br>* DL89: Pressure ulcer during or after admission.	During admission:<br>* Yes.<br>* No.	-	During admission:<br>* No pressure ulcer.<br>* Category 1: Persistent discoloration.<br>* Category 2: Epithelial damage with blisters, crack or abrasion of the skin.

Kommenterad [MH1]: during the hospital stay I suppose otherwise difficult

				* Category 3: Full-thickness skin defect without deep wound cavity.<br>* Category 4: Full-thickness skin defect with deep wound cavity.
**Urinary tract infection**	Available through ICD-10 code from the National Patient Registry: N08.0, N10, N11, N12, N13.6, N15.1, N15.9, N16.0, N28.8D–F, N29.0, N29.1, N30, N33.0, N34.0, N34.1, N39.0, etc., covering UTI during or after admission	During admission:<br>* Yes.<br>* No.	-	During admission:<br>* Yes.<br>* No.
**Pneumonia**	Available through ICD-10 code from the National Patient Registry: DSJ12–J18 for pneumonia, during or after admission.	During admission:<br>* Yes.<br>* No.	-	During admission:<br>* Yes.<br>* No.
**Acute kidney injury**	Available from the National Laboratory Registry. Can be calculated according to various definitions.	Acute kidney injury is defined as:<br>increase in serum creatinine by at least 50% or by 50 µmol/L from baseline.<br>Recorded as:<br>* yes.<br>* no.	-	Acute kidney injury is defined as:<br>increase of serum-Urea or Creatinine from initial reference point.<br>Recorded as:<br>* yes.<br>* no.
**Thrombosis**	Available through ICD-10 code from the National Patient Registry:<br>* DI80.1-3.<br>* DI26.<br>Either during or after admission.	During admission:<br>* Yes.<br>* No.	-	During admission:<br>* Yes.<br>* No.
**Cardiovascular event**	Available through ICD-10 code from the National Patient Registry:<br>* DI21,<br>* DI63-64,<br>* DG45.9,<br>Either during or after admission.	During admission.<br>Defined as myocardial infarction or stroke:<br>* yes.<br>* no	-	During admission:<br>* Yes.<br>* No.
**Delirium**	Available through ICD-10 code from the National Patient Registry:<br>* DR410: confusion.	During admission.<br>Delirium must be evaluated by 4AT or CAM:	-	During admission:<br>* yes,<br>* no.

	* DF05: delirium (not caused by alcohol or psychoactive substances).<br>* DF11: mental disorder or behavioral disorder caused by opioids.<br>Either during or after admission.	* yes,<br>* no		
**PROM**				
**Baseline and follow-up**	-	PROM evaluated at 4 times via EQ5D and HOOS-12.<br>* At baseline.<br>* At 4 months post surgery.<br>* At 1 year post surgery.<br>* At 3 year post surgery.	PROM evaluated at 2 times via EQ5D and SMFA.<br>* At baseline.<br>* At 1 year post surgery.	PROM evaluated once via EQ5D.<br>* At 4 months post surgery **

DMHFR = Danish Multidisciplinary Hip Fracture Registry, NHFR = Norwegian Hip Fracture Register, SFR = Swedish Fracture Registry, NAR = Norwegian Arthroplasty Registry, THA = Total Hip Arthroplasty
*** coverage and completeness uncertain*
